# Physics-Guided Variational Causal Intervention Network for Few-Shot Radar Jamming Recognition

**DOI:** 10.3390/s26061900

**Published:** 2026-03-18

**Authors:** Dong Xia, Liming Lv, Youjian Zhang, Yanxi Lu, Fang Li, Lin Liu, Xiang Liu, Yajun Zeng, Zhan Ge

**Affiliations:** 1 Graduate School of China Academy of Engineering Physics, Institute of Electronic Engineering, Mianyang 621900, China; 2 The Institute of Electronic Engineering, China Academy of Engineering Physics, Mianyang 621900, China

**Keywords:** radar jamming recognition, few-shot learning, physics-guided, causal inference

## Abstract

Rapid and accurate recognition of radar active jamming is a prerequisite for cognitive electronic countermeasures. However, under complex electromagnetic environments with scarce training samples, existing deep learning models are prone to capturing spurious correlations induced by environmental confounders, resulting in notable performance degradation. To address this causal confounding issue, we propose a physics-guided variational causal intervention network (PG-VCIN). First, we reconstruct a structured causal model of jamming signal generation, decoupling observations into robust physical statistical features and sensitive time–frequency image representations. Physical priors are then leveraged to perform dynamic precision-weighted modulation of visual feature extraction, enforcing physical consistency at the representation learning stage. Second, we formulate deconfounding within an active inference framework and introduce a variational information bottleneck to optimize mutual information, thereby filtering out high-complexity redundant information attributable to confounders while preserving the essential causal semantics. Finally, we numerically approximate the causal effect by imposing dual intervention constraints in the latent space, including intra-class invariance and confounder invariance. Experiments on a semi-physical simulation dataset demonstrate that the proposed method achieves substantially higher recognition accuracy than several representative few-shot baselines in extremely low-sample regimes, validating the effectiveness of integrating physical mechanisms with causal inference.

## 1. Introduction

Radar is a core component of modern sensing systems and plays an indispensable role in military reconnaissance, civil aviation, and meteorological monitoring [1,2]. By transmitting electromagnetic waves and processing the received echoes, radar enables target detection, localization, and tracking, with all-weather and day–night operational capability. Nevertheless, radar systems operate in contested electromagnetic environments. The rapid development of electronic countermeasures—particularly digital radio frequency memory (DRFM) technology—has posed severe threats in the form of deception jamming [3,4]. DRFM can accurately replicate and manipulate radar signals to synthesize highly realistic false targets, thereby disrupting normal radar operation and potentially inducing false alarms or missed detections.

To address this challenge, anti-jamming techniques have become a central focus in radar system design, where jamming recognition constitutes a key prerequisite for cognitive anti-jamming architectures [5,6]. Accurate identification of jamming types provides the decision basis for adaptively adjusting transmission parameters or selecting optimal countermeasure strategies, thus improving radar survivability in complex electromagnetic environments. More broadly, robust jamming recognition is also relevant to the protection of sensor-networked and distributed sensing systems, where intentional interference may degrade communication reliability and fusion performance [7,8].

The first route is expert-knowledge-driven feature engineering combined with conventional pattern recognition. Prior to the rise of deep learning, radar jamming recognition largely relied on signal processing and statistical learning. Discriminative features were manually crafted in the time, frequency, and time–frequency domains—such as energy distribution descriptors, texture statistics, and higher-order spectral measures—and then fed into classical classifiers, including support vector machines, random forests, and discriminant analysis. These methods are typically interpretable, but their performance depends strongly on handcrafted features and empirical rules, which limits robustness under cross-scenario parameter variations. Representative studies include deception jamming recognition based on product spectrum matrix (SPM) feature extraction [9], diagonal integral bispectrum-based detection and discrimination for DRFM deception jamming [4], and low-SNR recognition using multi-domain statistics [10]. In addition, rule-based feature construction and robust discrimination tailored to specific radar waveforms and deception modes have been continuously explored [11].

The second route is represented by deep learning. With powerful end-to-end representation learning, deep models substantially reduce reliance on handcrafted features and have driven notable advances in radar jamming recognition. Recent studies commonly input time–frequency representations of radar signals into convolutional networks, attention-based architectures, or multi-branch fusion frameworks to learn highly discriminative high-dimensional embeddings, achieving effective recognition of complex patterns such as deception and compound jamming [12,13,14,15]. For instance, Lan et al. exploited multi-pulse information fusion for intelligent recognition of chirp-radar deceptive jamming [12]; Li et al. improved compound-jamming recognition via time–frequency image segmentation and an attention residual network [13]; and Zhang et al. enhanced performance through time-domain weighted fusion with attention mechanisms [14]. Collectively, these results demonstrate clear advantages of data-driven models in complex jamming modeling and nonlinear discrimination.

However, idealized deep models typically assume access to large-scale, diverse, and high-quality labeled datasets, which substantially deviates from practical radar deployments. In operational or test-range scenarios, acquiring sufficient labeled jamming samples is constrained by multiple factors: jamming tactics and parameters evolve rapidly, while labeled data accumulation often lags behind operational needs; meanwhile, data collection costs, confidentiality restrictions, and labeling difficulty make sample scarcity the norm. Under such small-sample conditions, deep models are prone to overfitting and may learn spurious correlations associated with simulation settings, parameter perturbations, or noise statistics, leading to cross-scenario performance degradation.

Few-shot learning aims to enable models to rapidly acquire new concepts from only a few labeled examples. This line of research has developed into a relatively mature framework in computer vision and has been progressively introduced into radar jamming recognition to address the problem of data scarcity. Several studies have formulated radar jamming recognition under few-shot settings and proposed corresponding architectures or training strategies, including improving generalization under limited samples through multi-channel time–frequency construction, weighted voting, and transfer learning [16]; few-shot recognition networks that combine time–frequency self-attention with global knowledge distillation to balance global modeling and real-time capability [17]; PSPNet-based frameworks that integrate enhanced prototypical learning with pretraining and self-supervised fine-tuning for few-shot deception jamming recognition [18]; prototype-feature-driven strategies for few-shot active jamming recognition [19]; and few-shot recognition in the presence of unknown or open-set jamming types [20].

Despite substantial progress, a common limitation persists: insufficient attention to causal confounding. With limited data, received radar signals are inevitably contaminated by environmental factors (e.g., signal-to-noise ratio, clutter conditions, channel fluctuations, and platform motion errors). From a causal-graph perspective, these factors can act as confounders that simultaneously influence the input representations and the learned decision boundaries, making models more likely to capture spurious associations in few-shot regimes; when the test environment changes, these associations break down and recognition performance can deteriorate sharply. Related issues have been systematically discussed in limited-data SAR recognition, where dual-invariance intervention has been introduced to mitigate confounding effects [21]. On the other hand, compared with generic image classification, radar signals possess explicit mathematical models and physically grounded generation mechanisms. Such physical priors are often more robust to environmental variations and may serve as stable anchors or instrumental variables to guide feature extraction and inference. Nevertheless, existing approaches commonly fuse physical features and deep features via simple concatenation or weighting, lacking a unified mechanism that deeply embeds physical mechanisms into the causal inference chain. To better position the present work, representative related studies are summarized in Table 1.

Motivated by these observations, we propose a physics-guided variational causal intervention network (PG-VCIN) for robust inference in few-shot radar jamming recognition. Specifically, we (i) explicitly decouple observations into physical statistical features and time–frequency image representations and use physical priors to modulate the visual feature extraction process; (ii) introduce a variational information bottleneck in the latent space for compression and redundancy reduction, and incorporate dual-invariance intervention constraints to approximate causal intervention effects; and (iii) conduct comprehensive evaluations on a self-constructed dataset. The main contributions are summarized as follows:

1. A multimodal structured causal model for jamming recognition and an explanation of few-shot degradation. We decouple observations into physical features and time–frequency images and design a feature modulation mechanism in which physical priors dynamically reweight the precision of time–frequency feature extraction, enabling top-down cognitive regulation and deep multimodal fusion at the architectural level.
2. A physics-guided variational latent modeling and intervention learning framework. We develop a Feature-Wise Linear Modulation (FiLM)-based physical-prior modulation module for physically consistent multimodal fusion [22]; adopt a variational information bottleneck (VIB) to map features to probability distributions and optimize the mutual information objective, thereby suppressing noise and environmental bias; and further impose improved dual-invariance intervention constraints (an intra-class invariance proxy and a confounder invariance loss) in the latent space to approximate causal intervention effects with reduced computational cost.
3. Dataset construction and systematic validation. We build a dataset by combining model-based simulation with jammer emulation. Extensive ablation and comparative experiments are conducted on a dataset containing seven typical jamming types, validating the rationality and effectiveness of the proposed method. The results show that PG-VCIN significantly outperforms existing methods under 5-shot and 10-shot settings. Moreover, benefiting from the efficient compression of VIB and the structured guidance of physical mechanisms, the model size is substantially reduced, meeting real-time requirements for edge deployment.

The remainder of this paper is organized as follows. Section 2 presents the signal model of active radar jamming, feature construction, and a structured causal model incorporating environmental confounders and provides a theoretical analysis of the root causes of few-shot degradation. Section 3 details the PG-VCIN framework, including the physical-prior modulation module, variational information bottleneck modeling, and the objective functions and training procedure with dual-invariance intervention constraints. Section 4 describes dataset construction, experimental settings, baseline methods, evaluation metrics, and ablation studies and discusses cross-scenario generalization and lightweight deployment results. Finally, Section 5 concludes the paper and outlines future research directions.

## 2. Signal Modeling and Theoretical Analysis

### 2.1. Signal and Jamming Models

In this study, a linear frequency modulation (LFM) pulse is adopted as the radar transmitted waveform. Based on typical active jamming mechanisms reported in the literature, seven representative jamming patterns are selected for modeling and analysis, including range multi-false-target jamming (RMJ), velocity multi-false-target jamming (VMJ), chopping and interleaving (C&I) jamming, interrupted sampling repeater jamming (ISRJ), interrupted sampling and cyclic repeater jamming (ISCJ), smeared spectrum jamming (SMSP), and smart noise jamming (SNJ) [3,4,5,23,24,25,26]. These jamming types cover several representative mechanism classes, including dense false-target generation, interrupted sampling retransmission deception, spectral spreading, and signal-like noise modulation, thereby providing a diverse yet experimentally manageable basis for subsequent modeling and few-shot recognition analysis.

The transmitted LFM signal s(t) is expressed as

(1) $$s\left(t\right)=\exp(j\pi kt^{2}+j2\pi f_{0}t),$$

where $f_{0}$ denotes the carrier frequency, $k=\frac{B}{T}$ is the chirp rate, *B* is the signal bandwidth, and *T* is the pulse width.

RMJ generates dense false targets in range by producing multiple delayed replicas of the intercepted waveform:

(2) $$j_{\mathrm{RMJ}}\left(t\right)=\sum_{i=1}^{N}A_{i}s\left(t-\tau_{i}\right),$$

where *N* is the number of false targets, $\tau_{i}$ is the delay of the *i*-th false target, and $A_{i}$ is the amplitude weighting coefficient.

VMJ imposes multiple Doppler frequency shifts on the LFM signal to synthesize false targets with different apparent radial velocities:

(3) $$j_{\mathrm{VMJ}}\left(t\right)=\sum_{i=1}^{N}A_{i}s\left(t\right)e^{j2\pi \Delta f_{i}t},$$

where *N* is the number of false targets, $\Delta f_{i}=\frac{2v_{i}f_{0}}{c}$ is the Doppler frequency shift, $v_{i}$ is the false velocity, and *c* is the speed of light.

C&I segments the intercepted LFM pulse in time and then rearranges and reconstructs the segments to form deceptive echoes:

(4) $$j_{C\&I}\left(t\right)=\sum_{i=1}^{N^{\prime }}p\;\left(t-\frac{iT}{M^{\prime }N^{\prime }}\right),$$

with $p\left(t\right)=s\left(t\right)\left[\mathrm{rect}\;\left(\frac{t-\tau }{\tau }\right)\sum_{i=0}^{M^{\prime }-1}\delta \left(t-iT_{s}\right)\right],$ where $M^{\prime }$ is the number of rectangular pulse trains, $N^{\prime }$ is the number of padded segments per slice, $T_{s}$ is the fundamental period of the rectangular pulse train, and τ is the sampling pulse width. Here, rect(·) denotes the rectangular window and δ(·) denotes the Dirac delta function.

ISRJ periodically samples the intercepted signal with sampling period $T_{s}$ and sampling width τ and immediately retransmits the sampled fragments:

(5) $$j_{\mathrm{ISRJ}}\left(t\right)=\sum_{i=1}^{M}s\left(t\right)\cdot \mathrm{rect}\;\left(\frac{t-\frac{\tau }{2}-\left(i-1\right)T_{s}}{\tau }\right),$$

where *M* is the number of sampling operations, $T_{s}$ is the sampling period, and τ is the sampling pulse width.

ISCJ further introduces cyclic retransmission by repeatedly forwarding both the current and previously intercepted fragments, which leads to more complex deceptive structures:

(6) $$j_{\mathrm{ISCJ}}\left(t\right)=\sum_{i=0}^{M}\sum_{l=1}^{M-i+1}s\left(t-q_{il}\right)\cdot \mathrm{rect}\;\left(\frac{t-p_{i}\tau -q_{il}\tau }{\tau }\right),$$

where *M* is the number of sampling operations and τ is the sampling pulse width, with $p_{i}=\frac{i(i+1)-2}{2},\;q_{il}=\frac{(l-1)(2i+l+2)+2}{2}.$

SMSP spreads the signal spectrum through phase modulation, thereby altering the instantaneous frequency structure and broadening the spectral distribution:

(7) $$j_{\mathrm{SMSP}}\left(t\right)=\sum_{i=1}^{N}A_{i}\exp\;\left(j\pi k^{\prime }{\left(t-i\tau^{\prime }\right)}^{2}+j2\pi k^{\prime }\left(t-i\tau^{\prime }\right)\right),$$

where $\tau_{i}$ denotes the delay, $A_{i}$ is the amplitude weighting coefficient, $k^{\prime }=Nk$ is the chirp rate of the jamming signal, and $\tau^{\prime }=T/N$ is the jamming pulse width.

SNJ modulates noise into the signal subspace, yielding a jamming waveform with a signal-like structure while introducing stochastic perturbations:

(8) $$j_{\mathrm{SNJ}}\left(t\right)=s\left(t\right)\cdot n\left(t\right),$$

where n(t) denotes the modulated noise process.

### 2.2. Feature Extraction and Representation Construction

To comprehensively characterize deception jamming under limited data, each observation is represented by two complementary feature groups: (1) an STFT-based time–frequency image representation that preserves fine-grained structural patterns in the time–frequency plane; and (2) physics-informed statistical features that are directly related to signal generation mechanisms and statistical regularities and are typically more robust to environmental variations. This dual-view representation enables the model to exploit both expressive time–frequency details and physically reliable structural cues, providing a unified input for subsequent robust inference.

**Figure 1:**
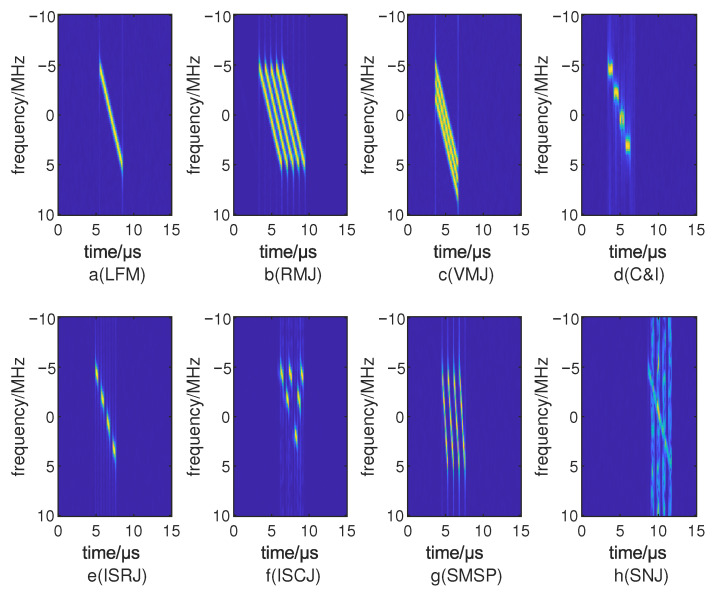
Time–frequency image.

Radar deception jamming is inherently non-stationary, and different jamming patterns exhibit distinguishable energy distributions over the joint time–frequency domain. The continuous short-time Fourier transform (STFT) is defined as

(9) $$S_{x}\left(\tau ,f\right)=\int_{-\infty }^{+\infty }x\left(t\right)\;w^{*}\left(t-\tau \right)\;e^{-j2\pi ft}\;dt,$$

where x(t) denotes the received complex baseband signal, w(t) is the analysis window, (τ,f) are the time and frequency coordinates, and ${\left(\cdot \right)}^{*}$ denotes complex conjugation. STFT offers a practical trade-off between time and frequency resolution and is well suited to describing the energy distribution of non-stationary signals. Compared with the Wigner–Ville distribution and wavelet transforms, STFT is computationally more efficient. Therefore, STFT is adopted as the time–frequency analysis tool in this work. The time–frequency image feature is constructed from the magnitude spectrum $|S_{x}\left(\tau ,f\right)|$ and fed into the visual branch of the network. Representative STFT time–frequency images of the signal and typical deception jamming patterns are illustrated in Figure 1.

In addition to time–frequency images, a 13-dimensional set of physics-informed statistical features is extracted. The key motivation is that, under few-shot conditions, models are more likely to be driven by environmental factors and thus capture spurious correlations, whereas physics-informed statistics often correspond to modulation structure, non-Gaussianity, higher-order phase coupling, multi-scale complexity, and energy concentration after coherent processing, which tend to be more stable and interpretable across environments. Accordingly, the physical features are organized into three levels to capture: (i) distributional shape and energy concentration, (ii) higher-order spectral coupling and multi-scale complexity, and (iii) coherent-processing-related discriminative cues for LFM waveforms. Specifically, the 13 features include time-domain skewness and kurtosis, and envelope fluctuation; frequency-domain skewness and kurtosis and a carrier-related factor; bispectrum mean and variance; wavelet-scale entropy and wavelet singular-spectrum entropy; the number of cepstrum peaks; de-chirp-domain kurtosis; and a kurtosis feature computed in the fractional Fourier transform (FrFT) domain [27,28,29,30].

For an arbitrary real-valued process u(t) with mean μ and standard deviation σ, the continuous skewness and kurtosis are defined as

(10) $$\mathrm{Skew}\left(u\right)=E\;\left[{\left(\frac{u(t)-\mu }{\sigma }\right)}^{3}\right],\;\mathrm{Kurt}\left(u\right)=E\;\left[{\left(\frac{u(t)-\mu }{\sigma }\right)}^{4}\right].$$

Let a(t)=|x(t)| be the signal envelope. The envelope fluctuation is quantified by the coefficient of variation

(11) $$\mathrm{EF}\left(x\right)=\frac{\sqrt{E\;\left[{\left(a\left(t\right)-E\left[a\left(t\right)\right]\right)}^{2}\right]}}{E[a(t)]}.$$

The frequency-domain moments are computed in the same manner on the magnitude spectrum A(f)=|X(f)|, yielding the frequency-domain skewness and kurtosis. The carrier-related factor characterizes the deviation in the spectral energy distribution from the carrier frequency. Using the spectral centroid $f_{c}$ and bandwidth *B*, it is defined as

(12) $$f_{c}=\frac{\int_{-\infty }^{+\infty }f\;A\left(f\right)\;df}{\int_{-\infty }^{+\infty }A\left(f\right)\;df},\;\mathrm{CF}=\frac{|f_{c}-f_{0}|}{B},$$

where $f_{0}$ denotes the carrier frequency.

For higher-order spectral features, the bispectrum is expressed as the third-order cumulant spectrum

(13) $$B\left(f_{1},f_{2}\right)=E\;\left\{X\left(f_{1}\right)\;X\left(f_{2}\right)\;X^{*}\left(f_{1}+f_{2}\right)\right\},$$

where X(f) is the Fourier transform of the signal. The bispectrum mean and variance are computed over the principal domain Ω of $\left|B(f_{1},f_{2})\right|$:

(14) $$\mu_{B}=\frac{1}{\left|\Omega \right|}\underset{\Omega }{\;\int \int \;}\left|B\left(f_{1},f_{2}\right)\right|\;df_{1}df_{2},\;\sigma_{B}^{2}=\frac{1}{\left|\Omega \right|}\underset{\Omega }{\;\int \int \;}{\left(|B\left(f_{1},f_{2}\right)|-\mu_{B}\right)}^{2}\;df_{1}df_{2}.$$

For multi-scale complexity, let $c_{j}\left(t\right)$ denote the wavelet coefficients at scale *j* and define the scale energy

(15) $$E_{j}=\int {\left|c_{j}\left(t\right)\right|}^{2}\;dt,\;p_{j}=\frac{E_{j}}{\sum_{j=1}^{J}E_{j}}.$$

The wavelet-scale entropy is then

(16) $$H_{\mathrm{ws}}=-\sum_{j=1}^{J}p_{j}\log\left(p_{j}\right).$$

Moreover, constructing a coefficient matrix from multi-scale wavelet coefficients and performing singular value decomposition yields singular values ${\left\{\lambda_{i}\right\}}_{i=1}^{r}$. Let $q_{i}=\lambda_{i}/\sum_{i=1}^{r}\lambda_{i}$. The wavelet singular-spectrum entropy is

(17) $$H_{\mathrm{wss}}=-\sum_{i=1}^{r}q_{i}\log\left(q_{i}\right).$$

For coherent-processing-related cues, the cepstrum is defined as

(18) $$c\left(\xi \right)=\left|F^{-1}\left\{\log\left(A(f)+\epsilon \right)\right\}\right|,$$

where ξ denotes the quefrency variable. The number of cepstrum peaks is used to characterize the richness of prominent peak structures in the cepstrum domain. The de-chirp operation is written as $y\left(t\right)=x\left(t\right)\;s_{\mathrm{ref}}^{*}\left(t\right)$, where $s_{\mathrm{ref}}\left(t\right)$ is a reference LFM signal; the de-chirp-domain kurtosis is computed on |y(t)| using (10). Finally, to describe the energy concentration behavior of LFM-like signals in a matched fractional order, we introduce FrFT-domain features. The α-order FrFT of x(t) is defined as

(19) $$X_{\alpha }\left(u\right)=\int_{-\infty }^{+\infty }x\left(t\right)\;K_{\alpha }\left(t,u\right)\;dt,$$

where for α≠nπ (n∈Z), the kernel is

(20) $$K_{\alpha }\left(t,u\right)=\sqrt{\frac{1-j\cot\alpha }{2\pi }}\;\exp\;\left(j\frac{t^{2}+u^{2}}{2}\cot\alpha -jtu\;\csc\alpha \right).$$

Based on the magnitude distribution $|X_{\alpha }\left(u\right)|$, the FrFT-domain kurtosis feature is computed using (10) to quantify energy concentration and distributional shape differences.

### 2.3. Causal Model, Latent Representation, and Intervention Objective

**Figure 2:**
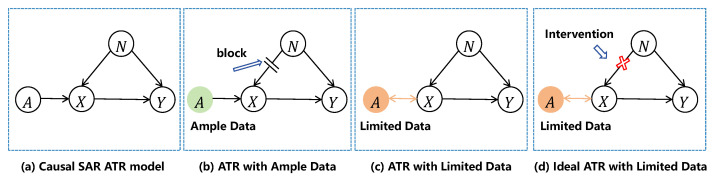
Causal SAR ATR model and the role of intervention under different data regimes. (**a**) Causal SAR ATR model with a confounder *N* affecting both the feature *X* and the predicted class *Y*, while the target attribute *A* influences *X* and *Y* along the causal path. (**b**) ATR with ample data, where the adverse impact of confounding can be partially suppressed by data coverage. (**c**) ATR with limited data, where the model tends to exploit the backdoor path induced by *N*, resulting in spurious correlations. (**d**) Ideal ATR with limited data, where an intervention blocks the influence of *N* on *X* to approximate the interventional effect.

When environmental factors simultaneously affect the observed representations and the classification outcome, correlation-based learning on observational data may capture confounding-induced spurious associations. Under few-shot settings, such spurious cues are more likely to be amplified, leading to pronounced performance degradation when the environment changes. Wang *et al.* systematically analyzed this issue in limited-data synthetic aperture radar automatic target recognition (SAR ATR) using a causal inference framework and advocated shifting from the observational distribution to an interventional one [21]. As illustrated in Figure 2, their causal graph introduces a confounder *N* that affects both the observation *X* (SAR image features) and the label *Y*, while the target attribute *A* influences *X* and *Y* along the causal path. With sufficient data, the negative effect of confounding can be partially suppressed by dataset coverage, and the learned effect P(Y∣X) may approximate the ideal interventional effect P(Y∣do(X)). In contrast, with limited data, the learned model tends to rely on the backdoor path induced by *N*, and explicit intervention is required to mitigate the confounding influence, which motivates the dual-invariance principle proposed in [21].

**Figure 3:**
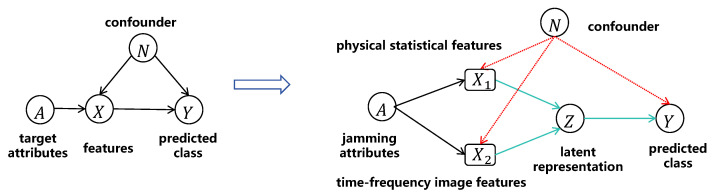
Causal graph adaptation from [21] to the proposed multimodal radar deception jamming recognition setting. The observation is decomposed into physical statistical features $X_{1}$ and time–frequency image features $X_{2}$, and a latent representation *Z* is introduced for multimodal fusion and intervention-motivated learning under confounding.

Following the causal modeling and intervention–derivation line in [21], we tailor the causal structure to radar deception jamming recognition by incorporating domain knowledge in radar signal processing and the multi-source nature of radar representations. Specifically, instead of a single observation variable *X* in [21], we explicitly represent the observation using two complementary feature modalities, as shown in Figure 3: $X_{1}$ denotes physical statistical features inspired by signal mechanisms, and $X_{2}$ denotes the time–frequency image representation obtained via STFT. Moreover, we introduce a latent representation variable *Z* as a unified intermediate space obtained by encoding $\left(X_{1},X_{2}\right)$, which facilitates multimodal fusion and enables intervention-motivated regularization in a common representation space. Let *A* denote the jamming modulation attributes (e.g., pulse width and Doppler shift) that govern the signal generation mechanism, *Y* denote the jamming-category label, and *N* denote the set of environmental factors accounting for variations such as SNR changes, clutter/background differences, channel fluctuations, and platform motion errors. The main causal relations are summarized as

(21) $$A\to X_{1},\;A\to X_{2},\;\left(X_{1},X_{2}\right)\to Z\to Y,$$

together with the confounding effects of the environment on both observations and outcomes:

(22) $$N\to X_{1},\;N\to X_{2},\;N\to Y.$$

Under this SCM, *N* may induce distribution shifts across conditions (captured by N→Y) and also alter the feature statistics and the latent representation formation (captured by $N\to X_{1},X_{2}\to Z\to Y$). With few labeled samples, limited coverage of environmental conditions makes such non-causal associations difficult to be averaged out, thereby deteriorating cross-environment generalization.

This issue can be understood from the mixture form of the predictive distribution. By the law of total probability, the observational predictive distribution is

(23) $$P\left(Y|X\right)=\sum_{N}P\left(Y|X,N\right)\;P\left(N|X\right),$$

where *X* denotes the observed features in general. In few-shot regimes, sampling bias yields an unstable estimate of P(N∣X), and the learned decision boundary can inadvertently absorb environment-specific bias. To suppress confounding paths, the backdoor criterion motivates the interventional distribution

(24) $$P\left(Y|do\left(X\right)\right)=\sum_{N}P\left(Y|X,N\right)\;P\left(N\right),$$

which replaces the environment-dependent weight P(N∣X) with the prior P(N), thereby correcting the environmental bias in a principled manner.

In practical radar applications, *N* is usually not fully observable nor precisely annotated. To make the intervention formulation tractable, we introduce the latent representation *Z* as a unified intermediate space: it facilitates multimodal fusion and regularization, and it also allows controlling representation complexity to reduce redundant details that are highly correlated with environmental variations. With the latent variable, using the observation exchange rule, the interventional distribution can be written in a form that involves *Z*:

(25) $$P\;\left(Y|do(X_{1},X_{2})\right)=\sum_{N}P\left(Y,Z|N\right)\;\frac{P(N)}{P(Z|N)}.$$

The ratio $\frac{P(N)}{P(Z|N)}$ acts as a bias-correction term: when the dependence of *Z* on *N* is reduced, P(Z∣N) becomes less sensitive to the environment, and the correction term varies less across conditions, leading to a more confounding-robust prediction.

Following the derivation in [21], (25) can be further expressed in an energy-based form:

(26) $$P\left(Y|do\left(X\right)\right)\propto \sum_{Z}\exp\;\left(E(y,z)\right)\cdot \frac{P(N)}{P(Z|N)},$$

where exp(E(y,z)) measures the compatibility between the class *y* and the latent representation *z*, and $\frac{P(N)}{P(Z|N)}$ accounts for confounding correction. Consequently, the intervention objective naturally decomposes into two complementary components: (i) enhancing the class representation compatibility to encourage compact within-class representations in the latent space; and (ii) reducing the dependence of *Z* on *N* to make P(Z∣N) as invariant as possible across environments (equivalently, weakening the statistical association between *Z* and *N*). Based on this decomposition, our training strategy builds upon the dual-invariance principle in [21] and adapts it to the proposed multimodal causal structure and the introduced latent representation *Z*, which together support robust recognition under few-shot and cross-environment conditions.

## 3. Proposed Approach

### 3.1. Overall Architecture and Workflow of PG-VCIN

**Figure 4:**
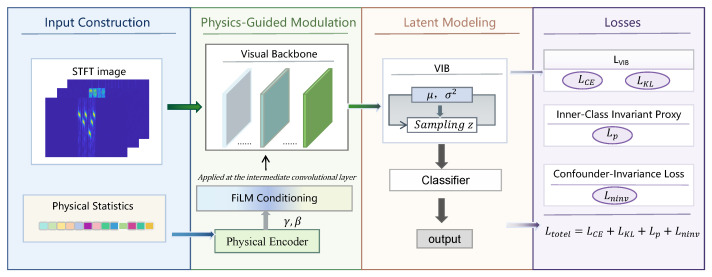
Overall architecture and training objectives of PG-VCIN.

This subsection presents the overall architecture and end-to-end workflow of the proposed PG-VCIN, as illustrated in Figure 4. PG-VCIN is developed for few-shot radar active deception jamming recognition. It targets robust classification under limited labeled data and varying operating conditions while maintaining a lightweight and deployable design. The framework integrates three key components. First, physical statistical features provide stable prior cues and condition deep feature extraction from time–frequency images. Second, VIB is introduced to compress the representation and suppress redundant information. Third, dual-invariance regularization is imposed to mitigate spurious correlations induced by environmental confounders and to improve cross-scenario generalization.

Given a received signal, a time–frequency image is constructed via STFT to preserve fine-grained structures of different jamming patterns. In parallel, a compact physical-feature vector is extracted from the signal to provide interpretable and relatively stable prior cues. The physical features are fed into a lightweight physical encoder to produce conditioning variables, which are injected into a designated intermediate stage of the visual backbone to update intermediate feature maps in a channel-wise manner through scaling and shifting. This conditional mechanism guides feature formation toward physically consistent time–frequency patterns and attenuates unstable variations caused by noise, clutter, and scenario-dependent parameter changes.

The time–frequency image branch adopts a lightweight CNN with six convolutional blocks. Each block consists of a 3×3 convolution (64 channels), BatchNorm, ReLU, and 2×2 max pooling. The visual backbone produces a fixed-dimensional embedding, which is then mapped by a lightweight projection head to obtain a unified representation for subsequent latent modeling and classification. On top of this representation, a VIB head parameterizes a latent distribution and produces a latent code via reparameterized sampling, which is fed into a lightweight classifier for jamming-category prediction. The distributional latent representation explicitly characterizes uncertainty under few-shot conditions. Meanwhile, the information bottleneck constraint suppresses redundancy correlated with environmental factors and provides a unified carrier for invariance regularization in the representation space.

Model optimization follows a K-shot class-balanced split. Specifically, a fixed number of samples per class are used for supervised training, and the remaining samples are further divided into validation and test sets for model selection and evaluation. The overall training objective combines a supervised classification loss, a VIB regularization term, and dual-invariance regularizers. The intra-class invariance term encourages same-class representations to concentrate around learnable class prototypes. The confounder invariance term constructs multiple pseudo-environments by grouping samples according to their similarity to class prototypes and penalizes environment-sensitive solutions through a gradient-based regularization. Subsequent sections detail the core modules and optimization. Section 3.2 describes physics-guided conditioning in the visual backbone. Section 3.3 presents VIB-based latent modeling and the information bottleneck regularization. Section 3.4 provides the complete objective function and the training procedure.

### 3.2. Physics-Guided Modulation

The physics-prior conditioning mechanism in PG-VCIN corresponds to the physical-feature encoder and the FiLM modulation module injected into the visual backbone. The design introduces stable prior cues carried by handcrafted physical statistical features during deep feature formation, so that the network is encouraged to learn mechanism-related and stable discriminative patterns under few-shot and environmentally perturbed conditions, while suppressing unstable appearance variations caused by noise, clutter, and scenario-dependent parameter changes.

Let the physical statistical feature vector be denoted by $x_{1}$. The physical branch applies a lightweight physical encoder to nonlinearly map $x_{1}$ and generate the channel-wise modulation coefficients γ and β for FiLM. In our implementation, the encoder is composed of stacked fully connected transformations with nonlinear activation and normalization, which improves representational capacity and optimization stability. As a result, the physical priors are converted into learnable, generalizable channel-level modulation signals. The FiLM module then broadcasts γ and β to the spatial dimensions and performs conditional updates on an intermediate feature map in the visual backbone.

Let $F\in R^{C\times H\times W}$ denote the intermediate feature map at the modulation location in the visual backbone, where *C*, *H*, and *W* are the number of channels and the spatial resolution, respectively. FiLM applies channel-wise scaling and shifting to F as

(27) $$\tilde{F}=F⊙\left(1+\gamma \right)+\beta ,$$

where ⊙ denotes channel-wise multiplication. The use of 1+γ keeps the modulation close to an identity mapping at early training stages, which improves optimization stability and convergence. In our implementation, the conditioning is applied at the output of the second convolutional block, enabling the physical prior to intervene at an early stage of feature formation and to influence subsequent hierarchical abstraction and aggregation of time–frequency structures.

From a mechanistic perspective, the scaling coefficients adjust the response strength of each channel, amplifying channels that are consistent with the physical priors while suppressing channels that are sensitive to environmental disturbances. The shifting coefficients modify the baseline activation of each channel, reshaping the feature distribution in a conditional manner. Together, they guide representation learning toward physically consistent time–frequency patterns, reduce reliance on noise-related textures and incidental scenario correlations, and alleviate overfitting under few-shot conditions.

After modulation, the visual backbone proceeds with subsequent convolution and pooling operations and outputs a fixed-dimensional representation for downstream projection, latent-space modeling, and classification. Since the physical prior acts directly on the feature formation process through conditioning, the resulting representation becomes less sensitive to environmental variations, providing a more robust input for the next section, where VIB is introduced for compression and de-redundancy and further invariance regularization is imposed in the representation space.

### 3.3. Variational Information Bottleneck for Robust Latent Learning

PG-VCIN performs latent-space modeling with VIB, following the standard information-theoretic formulation for compressed and robust representation learning [31]. The core purpose of this module is to probabilistically model the representation in the few-shot regime and to enforce compression and de-redundancy through an information bottleneck constraint, thereby reducing the impact of non-stationary information introduced by environmental factors on discrimination and providing a more robust representation for subsequent invariance regularization. Unlike conventional deterministic embeddings, VIB treats the network output as a parameterized distribution of a random variable, enabling the model to explicitly characterize representational uncertainty caused by data scarcity and to suppress redundancy irrelevant to class discrimination within a unified optimization form.

Specifically, let h denote the representation produced by the visual backbone and the projection head. The VIB head maps h to the parameters of a latent distribution, which is modeled as a diagonal Gaussian. Denote the mean and variance parameters by μ(h) and $\sigma^{2}\left(h\right)$, respectively, which define a conditional posterior distribution q(z∣h). To enable end-to-end training, the reparameterization trick is used to draw a latent code z from q(z∣h), and z is fed into the classifier for jamming-category prediction. Since latent sampling reflects representation stochasticity, the distributional formulation can alleviate instability caused by overfitting in few-shot training and yield more robust discriminative representations at inference time.

From an optimization perspective, VIB achieves compression and de-redundancy by minimizing the amount of information carried by the latent code while maintaining discriminability. This is typically implemented by jointly minimizing a supervised classification loss and a Kullback–Leibler (KL) divergence term between the latent posterior and a prior distribution. Intuitively, the classification loss encourages the latent code to preserve class-relevant discriminative information, whereas the KL regularizer limits extra information in the latent code. In this way, VIB enforces a representation-level constraint that retains essential information while filtering out nuisance factors, which is beneficial for improving stability under cross-scenario variations. The next subsection presents the overall objective function that integrates the classification loss, the VIB regularizer, and dual-invariance regularization, together with the corresponding training procedure.

### 3.4. Model Optimization and Training Procedure

This subsection presents the training objective and optimization strategy of PG-VCIN. Under few-shot conditions, environmental factors are more likely to act as confounders, and the model can easily rely on spurious correlations, leading to cross-scenario degradation. To address this issue, we incorporate a dual-invariance intervention loss in the representation space and jointly optimize it with the VIB regularization. The dual-invariance intervention, following [21], is composed of two complementary components. The first component enforces class-conditional stability to support reliable estimation of class structure under limited data, and the second component suppresses confounding effects by enforcing invariance across pseudo-environments constructed without explicit environment labels.

In PG-VCIN, *f* denotes the discriminative embedding fed to the classifier; when VIB is enabled, f=z, otherwise *f* is the projected feature before the classifier. Let $P_{i}$ be a learnable inner-class invariance proxy for class *i*. The inner-class invariance is encouraged by the proxy loss $L_{p}$, which pulls samples toward their class prototype while suppressing inter-class prototype confusion. Its key form can be written as

(28) $$L_{p}=-\sum_{i}\sum_{k}\lambda_{ik}\;d\;\left(\ell_{2}\left(f_{ik}^{w}\right),\ell_{2}\left(P_{i}\right)\right)\;+\;\sum_{i}\sum_{j}d\;\left(\ell_{2}\left(P_{j}\right),\ell_{2}\left(P_{i}\right)\right)+2,$$

where $\lambda_{ik}$ denotes an instance weight, $f_{ik}^{w}$ is the spatially reweighted feature, $\ell_{2}\left(\cdot \right)$ denotes $\ell_{2}$ normalization, and d(·,·) is a similarity metric. For confounder invariance, when environment labels are unavailable, non-anchor samples are partitioned into $K_{n}$ pseudo-environments according to a virtual confounding measure. Under the *n*-th pseudo-environment $\phi_{n}$, the relative distribution measure for anchor class *i* is defined as

(29) $$L_{di}^{\phi_{n}}=-\sum_{k}\log\frac{\exp(dv\left(f_{ik},P_{i}\right))}{\exp\left(dv\left(f_{ik},P_{i}\right)\right)+\sum_{f_{jl}\in D_{\neg A}^{\phi_{n}}}\exp\left(dv\left(f_{jl},P_{i}\right)\right)},$$

where $D_{\neg A}^{\phi_{n}}$ denotes the set of non-anchor samples in the *n*-th pseudo-environment, and dv(·,·) is the virtual confounding measure. To enforce risk consistency across pseudo-environments, an Invariant Risk Minimization (IRM)-style gradient penalty is added and summed over all anchor classes and pseudo-environments:

(30) $$L_{ninv}=\sum_{i=1}^{C}\sum_{n=1}^{K_{n}}\left(L_{di}^{\phi_{n}}+∥\nabla L_{di}^{\phi_{n}}{∥}_{2}^{2}\right).$$

All symbol definitions, construction details, and derivations of the dual-invariance terms strictly follow Wang et al. [21].

Beyond dual-invariance, VIB regularization is adopted to compress representations and suppress redundancy from an information-theoretic perspective. VIB parameterizes the latent variable z using a variational posterior $q_{\phi }\left(z|h\right)$ and a prior p(z), typically chosen as a standard normal distribution. The information bottleneck objective aims to maximize I(z;y) while minimizing I(z;h). The mutual information term admits the variational upper bound

(31) $$I\left(z;h\right)=E_{p(h)}\mathrm{KL}\;\left(q_{\phi }\left(z|h\right)\;∥\;q_{\phi }\left(z\right)\right)\leq E_{p(h)}\mathrm{KL}\;\left(q_{\phi }\left(z|h\right)\;∥\;p\left(z\right)\right),$$

where the marginal $q_{\phi }\left(z\right)$ is approximated by the chosen prior p(z). Maximizing I(z;y) can be implemented by minimizing the negative log-likelihood. Consequently, the variational information bottleneck objective is given by

(32) $$L_{\mathrm{VIB}}=E_{q_{\phi }\left(z|h\right)}[-\log p_{\theta }\left(y|z\right)]+\beta \;E_{p(h)}\mathrm{KL}\;\left(q_{\phi }\left(z|h\right)\;∥\;p\left(z\right)\right),$$

For implementation convenience, we write

(33) $$L_{\mathrm{VIB}}=L_{\mathrm{ce}}+L_{\mathrm{KL}},$$

where the standard cross-entropy loss is

(34) $$L_{\mathrm{ce}}=\frac{1}{B}\sum_{i=1}^{B}\mathrm{CE}\;\left({\mathrm{logits}}_{i},y_{i}\right),$$

and the KL regularization term is defined as

(35) $$L_{\mathrm{KL}}=\beta \cdot \frac{1}{B}\sum_{i=1}^{B}\mathrm{KL}\;\left(q_{\phi }\left(z_{i}|h_{i}\right)\;∥\;p\left(z\right)\right),$$

with β controlling the compression strength. This term limits the information content of latent representations, suppresses redundancy correlated with environmental factors, and mitigates instability caused by overfitting under few-shot conditions. The final optimization objective is written as

(36) $$L_{\mathrm{total}}=L_{\mathrm{ce}}+L_{p}+L_{\mathrm{ninv}}+L_{\mathrm{KL}}.$$

During training, PG-VCIN computes the supervised classification loss, the VIB KL regularizer, the intra-class invariance proxy, and the confounder invariance loss for each mini-batch and updates all parameters end-to-end. Following [21], the dual-invariance part is kept in its original unweighted form, whereas the compression strength of the KL regularizer is controlled by β. A sensitivity analysis with respect to β is further provided in Section 4.5. The training set is constructed by a class-balanced *K*-shot sampling strategy, and the remaining samples are split into validation and test sets for model selection and generalization evaluation. The effectiveness of the dual-invariance intervention and the VIB regularization under few-shot and cross-scenario conditions is further validated through ablation and comparative experiments in Section 4.

## 4. Experiments and Results

To comprehensively evaluate the performance of PG-VCIN on the few-shot recognition task of radar active deception jamming, a set of systematic experiments are conducted in this section. First, Section 4.1 details the dataset construction, experimental settings, and evaluation protocol. Next, Section 4.2 introduces the baseline methods and evaluation metrics. Then, Section 4.3 reports and analyzes the main comparative results. Section 4.4 conducts ablation studies to investigate the contributions of the core modules, verify the sources of performance gains, and explain the underlying mechanisms. Section 4.5 presents a sensitivity analysis of the VIB regularization coefficient. Finally, Section 4.6 provides a preliminary evaluation under compound-jamming conditions.

### 4.1. Dataset Construction and Experimental Settings

To ensure both diversity and realism, the radar active deception jamming dataset was constructed by combining model-based simulation with jammer emulation. As described in Section 2.1, the data generation adopts an LFM pulse as the reference transmitted waveform and synthesizes seven representative deception jamming types, including RMJ, VMJ, C&I, ISRJ, ISCJ, SMSP, and SNJ. The dataset contains nine classes in total: the above seven jamming types, the LFM echo class without jamming, and an RMJ class collected from a jammer simulator (denoted as RMJ*). The RMJ* class is introduced as a semi-physical category to provide a preliminary evaluation of cross-domain adaptability between purely simulated data and hardware-in-the-loop signal generation, thereby offering an intermediate step toward real-world deployment assessment. For each jamming type, key parameters are randomly sampled within typical operating ranges to reflect the uncertainty of jammer configurations in practice; the parameter ranges are summarized in Table 2. Each class contains 500 samples. Additive white Gaussian noise (AWGN) is added to the received complex baseband signal to emulate noisy electromagnetic environments. The signal-to-noise ratio (SNR) of each sample is independently and uniformly sampled from −15 dB to 10 dB, while the jammer-to-signal ratio (JSR) is fixed at 3 dB. In the current dataset, environmental variability mainly comes from AWGN corruption, random parameter perturbations of different jamming modes, and the domain discrepancy introduced by the semi-physical jammer-simulator samples, whereas structured environmental clutter is not explicitly included.

Experiments follow the standard *N*-way *K*-shot evaluation protocol in few-shot learning. In each training and testing episode, all N=8 simulated classes are included, and *K*-labeled samples per class are provided to form the support set for model adaptation. Additional samples from the same episode are used as the query set to evaluate classification performance. We first focus on the 20-shot setting (i.e., 20 training samples per class) and further investigate the more challenging 5-shot and 10-shot settings. To reduce the variance caused by random episode sampling, all reported results are averaged over 10 independent runs and reported in the form of mean ± standard deviation.It should also be clarified that the current few-shot protocol already involves a certain degree of parameter-level generalization within the predefined operating ranges. In each run, only *K*-labeled samples per class are used for training, so the support set covers only a very limited subset of the possible parameter realizations and SNR conditions. The validation and test sets are constructed from the remaining samples and therefore naturally include many instances of the same jamming type with parameter settings not present in the training set. Thus, the reported results reflect generalization to unseen same-class parameter instances within the predefined ranges, although they do not constitute a strict parameter-disjoint extrapolation study.

All experiments are conducted on a workstation equipped with an NVIDIA GeForce RTX 4070 Ti SUPER GPU (NVIDIA Corporation, Santa Clara, CA, USA). The implementation is based on Python 3.12 and PyTorch 2.5.1. The model is trained using the Adam optimizer with an initial learning rate of $1\times 10^{-3}$, and the KL regularization term is weighted by $\beta =10^{-3}$.

### 4.2. Baseline Methods and Evaluation Metrics

To ensure a comprehensive and fair assessment of the proposed PG-VCIN, this subsection presents the baseline methods and the evaluation metrics adopted in our experiments.

We reproduce four representative state-of-the-art approaches that have been widely used in radar jamming recognition, particularly under few-shot settings, including WECNN-TL [16], MAML [32], JR-TFSAD [17], and PSPNet [18].

To evaluate classification performance from multiple perspectives, we report four standard metrics, namely accuracy, recall, precision, and F1-score. Let TP, TN, FP, and FN denote the number of true positives, true negatives, false positives, and false negatives, respectively. These metrics are defined as

(37) $$\mathrm{Accuracy}=\frac{TP+TN}{TP+TN+FP+FN},$$

(38) $$\mathrm{Recall}=\frac{TP}{TP+FN},$$

(39) $$\mathrm{Precision}=\frac{TP}{TP+FP},$$

(40) $$F1=\frac{2\;\mathrm{Precision}\times \mathrm{Recall}}{\mathrm{Precision}+\mathrm{Recall}}.$$

Beyond accuracy-related metrics, computational efficiency and resource consumption are critical for practical deployment in real radar systems. Therefore, we additionally report model complexity in terms of floating-point operations (FLOPs) and the number of parameters (Params). FLOPs measure the computational cost of a single forward inference, reflecting the time complexity, while Params quantify the storage requirement of the model, reflecting the space complexity.

### 4.3. Main Results and Analysis

This subsection systematically reports and analyzes the jamming recognition performance of the proposed PG-VCIN and the baseline methods under typical few-shot settings. The experimental results are discussed from multiple perspectives, including overall performance, class-wise characteristics, robustness under different sample sizes, stability under different signal-to-noise ratio (SNR) conditions, and model complexity, thereby validating the effectiveness and advancement of the proposed method.

Under the 20-shot setting, the per-class accuracies over nine signal classes and the averaged performance metrics are summarized in Table 3 and Table 4, respectively. Overall, PG-VCIN achieves the best or near-best performance on most classes. In terms of averaged metrics, PG-VCIN substantially outperforms the baselines, reaching 97.77% accuracy, 97.76% recall, 97.75% precision, and 97.82% F1-score, which demonstrates its strong overall classification capability. The superiority of PG-VCIN is mainly attributed to the tight integration of physics-guided learning and causal intervention. Specifically, physical priors provide stable and interpretable feature anchors, while VIB and dual-invariance constraints effectively suppress spurious correlations induced by limited samples and environmental variations, encouraging the model to learn more generalizable causal representations. In comparison, although WECNN-TL and MAML are competitive to some extent, the former tends to overfit under extremely limited data due to its relatively high model complexity, whereas the latter exhibits noticeable performance fluctuations caused by the instability of meta-learning. JR-TFSAD performs well on certain jamming types such as ISRJ and SMSP, benefiting from its attention mechanism in capturing salient time–frequency structures; however, its overall performance varies considerably, indicating insufficient generalization to some complex jamming patterns. PSPNet achieves stable second-best performance due to the simplicity of metric learning and the advantage of pretraining, yet the performance gap of PG-VCIN becomes larger on the most confusing classes such as ISCJ and SNJ. This suggests that, for complex scenarios with large intra-class variation and high inter-class similarity, metric learning alone is still insufficient, and a more fundamental causal feature disentanglement mechanism is required. It is also worth noting that PG-VCIN maintains perfect recognition accuracy on the semi-physical RMJ* class under the 20-shot setting. This result provides preliminary evidence that the proposed framework can preserve discriminative capability under a certain degree of domain discrepancy between model-based simulation and jammer-simulator data. Several entries in Table 3 are rounded to 100.00%, mainly for classes such as RMJ, VMJ, and RMJ*, whose signatures are relatively distinctive under the current dataset composition and evaluation protocol. In particular, dense false-target jamming patterns and the semi-physical RMJ* samples exhibit comparatively stable and separable structures in the adopted representations, which makes near-saturated classification performance more likely in the 20-shot setting. By contrast, classes such as ISCJ and SNJ remain more challenging because of their higher structural complexity and stronger similarity to other interference patterns.

To further evaluate robustness under more severe data scarcity, Table 5 and Table 6 report the averaged performance under the 10-shot and 5-shot settings, respectively. As the number of training samples decreases from 20-shot to 5-shot, all methods exhibit performance degradation, which is consistent with the general behavior in few-shot learning. Nevertheless, PG-VCIN shows the strongest robustness, with the smallest performance drop. Even in the challenging 5-shot setting, PG-VCIN still achieves 91.84% accuracy, which is markedly higher than the competing methods. This observation highlights the intrinsic advantage of PG-VCIN in addressing the essence of data scarcity. By constraining the information channel, VIB forces the model to discard high-entropy and non-essential details in the input while preserving low-entropy causal semantics that are most relevant for class discrimination. Such an inherent denoising and purification mechanism makes PG-VCIN less prone to overfitting spurious statistical associations that occur accidentally in the limited training set. In contrast, MAML and JR-TFSAD suffer more severe degradation under 5-shot, indicating a stronger dependence on training-specific patterns and weaker generalization. PSPNet remains relatively stable under 5-shot, but the absolute performance gap to PG-VCIN becomes larger than that under 20-shot, implying that physics guidance and causal intervention become increasingly critical when supervision is extremely limited.

**Figure 5:**
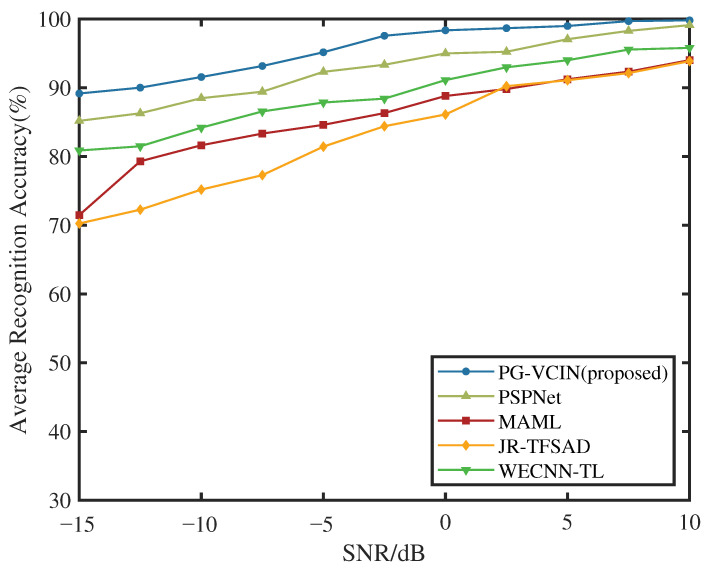
Average recognition accuracy versus SNR for different methods.

Beyond sample size, stability under different SNR conditions is an important criterion for practical applications. As shown in Figure 5, since the SNR in our dataset varies from −15 dB to 10 dB, low-SNR conditions significantly blur time–frequency structures and strengthen environmental confounding, making models more likely to learn non-causal patterns correlated with noise statistics. Therefore, we further analyze the recognition performance across different SNR ranges and focus on the degradation trend in low-SNR regimes. The results show that, although the accuracy of all methods decreases as SNR decreases, PG-VCIN exhibits a smaller degradation magnitude, indicating better noise robustness. This is mainly because physical statistical features can still provide relatively stable priors under low SNR, alleviating the representation collapse caused by relying solely on time–frequency images. Meanwhile, VIB suppresses non-essential details in high-noise backgrounds by limiting the information flow in the latent space, and the dual-invariance constraints further restrict the model from exploiting spurious correlations induced by SNR variations, thereby stabilizing the decision boundary under SNR fluctuations. In contrast, end-to-end models primarily driven by time–frequency images are more likely to suffer from class confusion under low SNR, especially for SNJ and ISCJ, which are strongly coupled with noise/modulation structures, reflecting higher sensitivity to environmental disturbances.

Model complexity comparison provides another important perspective. As shown in Table 7, while achieving the highest recognition accuracy, PG-VCIN maintains a parameter size and computational cost that are on the same order of magnitude as the most lightweight baselines and are substantially lower than those of the more complex WECNN-TL and MAML. This indicates that PG-VCIN achieves an efficient trade-off between performance and complexity. The performance gain is not achieved by simply increasing model capacity; instead, it benefits from the structured design guided by physical priors and the efficient utilization of information enabled by variational causal intervention. With relatively low computational overhead, PG-VCIN attains more robust and generalizable recognition performance, showing strong potential for deployment on resource-constrained edge devices.

In summary, the comparative experiments consistently confirm the comprehensive advantages of PG-VCIN for few-shot radar jamming recognition. The proposed method achieves the highest recognition accuracy under both moderate and extremely limited sample regimes, while maintaining low model complexity and strong robustness. These results validate the effectiveness and necessity of deeply integrating physical priors with causal inference mechanisms to address pattern recognition challenges in few-shot and noisy radar environments.

### 4.4. Ablation Study

To thoroughly investigate the contributions of the core components in the proposed PG-VCIN and their interactions, we conduct systematic ablation studies. Following the principle of controlling variables, key modules are progressively removed or replaced, and the performance variations are evaluated under the 20-shot setting. This enables a quantitative assessment of the effects of physics-guided modulation, VIB, and the dual-invariance constraints on the final recognition performance.

The ablation study is designed to isolate and evaluate three components. The baseline model only contains the dual-invariance intervention mechanism and serves as the reference for comparison. The physics-guided modulation module is introduced to validate the role of physical priors in feature fusion; when this module is removed, the model degrades to a simple two-stream feature concatenation followed by the variational head. The VIB module is used to verify the effectiveness of distributional latent modeling and information compression; when it is removed, the variational head is replaced by a standard fully connected layer, producing deterministic feature vectors instead of distribution parameters. The averaged recognition performance of different module combinations is reported in Table 8, showing that each component consistently improves the performance.

**Figure 6:**
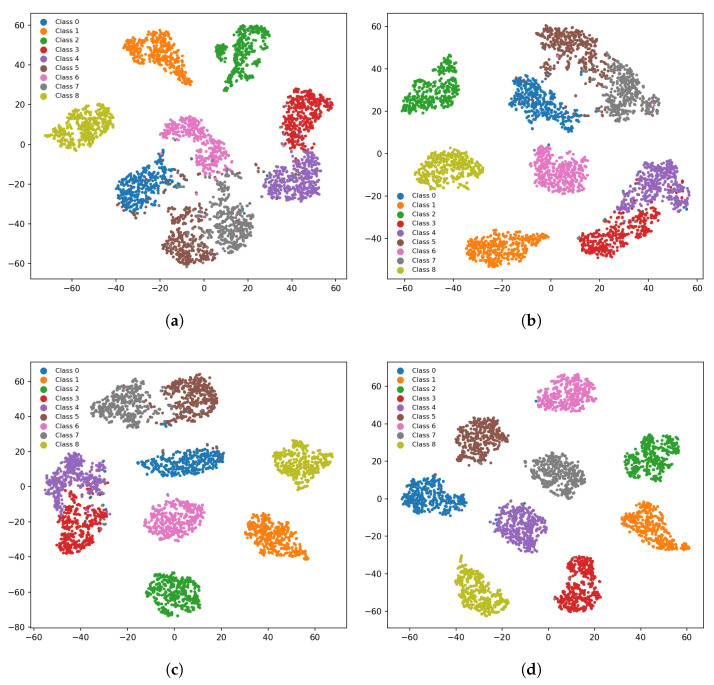
t-SNE visualization of latent representations for ablation variants under the 20-shot setting. (**a**) Baseline model with dual-invariance constraints only. (**b**) Baseline + VIB. (**c**) Baseline + physics-guided modulation. (**d**) Full model.

To visually illustrate how incremental module integration reshapes the feature space, we further provide t-SNE visualizations of the latent representations produced by four model variants on the test set. As shown in Figure 6, the baseline model already exhibits a certain clustering tendency, indicating that the invariance constraints are beneficial; however, the intra-class compactness is limited and the inter-class boundaries remain ambiguous. After incorporating physical priors, the clusters become more regular and the intra-class consistency is noticeably enhanced. With the compression effect of the information bottleneck, the clusters become more cohesive and the inter-class margins increase, resulting in a clearer separation. When both physics guidance and VIB are integrated, the latent space achieves the most desirable structure, with highly compact intra-class clusters and the largest inter-class separability, thereby enabling high-accuracy classification.

When neither physics-guided modulation nor VIB is used, the model achieves 91.84% accuracy and a 91.31% F1-score, which is close to the best-performing baseline in Section 4.3. This confirms the effectiveness of the core idea of approximating causal intervention via dual-invariance constraints: the invariance losses encourage learning robust features that are less dependent on environmental confounders, providing a fundamental guarantee for few-shot recognition. Nevertheless, the performance ceiling of the baseline also suggests that relying solely on loss-level constraints, without architectural guidance and purification of the feature generation process, yields limited deconfounding capability. Introducing VIB alone leads to a substantial improvement across all metrics, strongly demonstrating its value. The built-in information compression and purification effectively mitigates the tendency of deep models to overfit training-specific noise patterns under limited samples, thereby enhancing generalization and robustness. Similarly, introducing physics guidance alone also brings a clear performance gain, highlighting the benefit of embedding domain knowledge into deep networks. By incorporating expert knowledge in a differentiable and parameterized manner, physics guidance provides a stable and interpretable inductive bias. Finally, when physics guidance and VIB are jointly integrated, the model attains the best performance. These two components complement each other from different aspects, enabling the model to better approximate the underlying causal features and thus achieve superior recognition accuracy and robustness under extremely limited data.

### 4.5. Sensitivity Analysis of the VIB Regularization Coefficient

In the proposed PG-VCIN, the dual-invariance part of the objective follows the original formulation in [21], whereas the newly introduced variational regularization strength is controlled by the coefficient β. To evaluate the influence of this additional hyperparameter, we analyze the sensitivity of the model to different values of β under the 20-shot setting.

Specifically, β is varied in $\left\{10^{-4},5\times 10^{-4},10^{-3},5\times 10^{-3},10^{-2}\right\}$, and the corresponding averaged recognition performance is reported in Table 9. The results show that PG-VCIN is relatively stable within a moderate range of β, and the best performance is achieved near $\beta =10^{-3}$. When β is too small, the compression effect of VIB becomes insufficient; when it is too large, excessive compression may suppress discriminative information.

### 4.6. Preliminary Evaluation Under Compound-Jamming Conditions

Although the main benchmark in this work is formulated as a single-label few-shot recognition task, realistic electromagnetic environments may involve the coexistence of multiple jamming components. To further examine the behavior of PG-VCIN under more complex interference conditions, we additionally conduct a supplementary experiment under compound-jamming conditions.

According to the mechanism-based categorization discussed in Section 2.1, four representative jamming patterns are selected, namely RMJ, ISRJ, SMSP, and SNJ, which respectively correspond to representative false-target generation, sampling/retransmission-based deception, spectral spreading, and signal-like noise modulation mechanisms. Based on these four representative jamming types, six dual-jamming combinations are constructed by pairwise superposition, namely RMJ + ISRJ, RMJ + SMSP, RMJ + SNJ, ISRJ + SMSP, ISRJ + SNJ, and SMSP + SNJ. In this supplementary experiment, each dual-jamming combination is treated as a composite class, and the resulting task is formulated as an additional six-class recognition problem.

**Figure 7:**
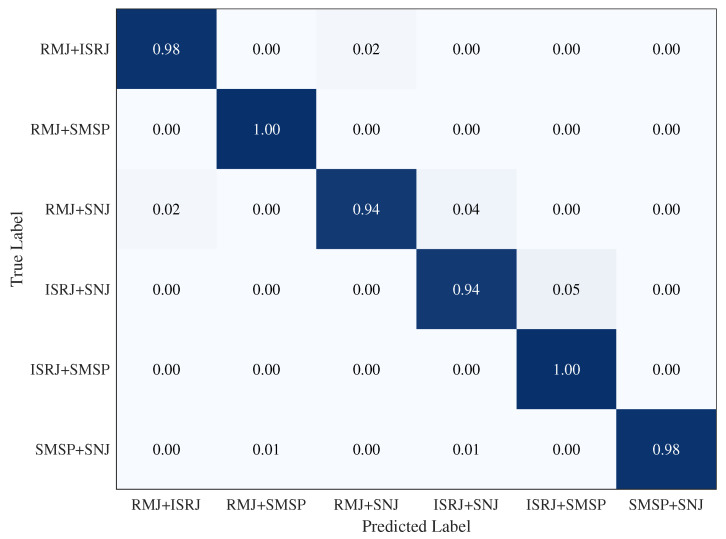
Confusion matrix of PG-VCIN under compound-jamming conditions in the supplementary 20-shot experiment.

Unless otherwise specified, the remaining experimental settings are kept consistent with those used in the main 20-shot experiments. The confusion matrix of PG-VCIN under compound-jamming conditions is shown in Figure 7, and the corresponding average performance metrics are summarized in Table 10.

PG-VCIN achieves 97.20% accuracy, 97.23% recall, 97.21% precision, and 97.20% F1-score under the supplementary 20-shot compound-jamming setting, indicating strong recognition capability even when multiple interference mechanisms coexist.

As shown in Figure 7, PG-VCIN maintains high recognition accuracy for most compound-jamming classes. In particular, the classes RMJ + SMSP and ISRJ + SMSP are recognized almost perfectly, while RMJ + ISRJ and SMSP + SNJ also achieve very high recognition rates. By contrast, RMJ + SNJ and ISRJ + SNJ are relatively more challenging, with limited confusion observed between RMJ + SNJ and ISRJ + SNJ, as well as between ISRJ + SNJ and ISRJ + SMSP. This phenomenon suggests that the introduction of a noise-like component tends to increase the structural complexity of the composite representation and may partially weaken the separability between certain jamming mixtures. Nevertheless, the overall results indicate that PG-VCIN preserves strong discriminative capability under the considered compound-jamming conditions, providing a useful preliminary step toward more complex interference-scene recognition.

## 5. Conclusions

This paper addressed few-shot radar active jamming recognition in complex electromagnetic environments, focusing on the key issue that sample scarcity and environmental variations can drive deep models to learn spurious correlations and thus fail to generalize across scenarios. To this end, a physics-guided variational causal intervention network was proposed. From a mechanism-informed perspective, PG-VCIN decouples observations into time–frequency image representations and handcrafted physical statistical features and performs physically consistent multimodal fusion via physics-guided modulation. Moreover, a variational information bottleneck is introduced in the latent space to compress redundant factors and suppress noise and environment-induced biases, while dual-invariance intervention regularization is incorporated to approximate deconfounded causal inference, thereby improving robustness under extremely limited samples. Comprehensive comparative experiments and ablation studies on a self-constructed semi-physical simulation dataset demonstrate that PG-VCIN achieves superior recognition performance under 5-shot and 10-shot settings and simultaneously exhibits favorable lightweight characteristics in terms of parameter size and computational cost, meeting the real-time requirements of edge deployment. Future work will further validate the proposed framework under more challenging electromagnetic conditions and broader radar operating configurations. In particular, structured environmental clutter and fully measured real-world radar data will be incorporated to assess transferability under more realistic deployment conditions. In addition, more general compound-jamming scenarios and cross-parameter generalization will be investigated to further evaluate the robustness of the proposed method in complex interference environments. Further effort will also be devoted to improving deployment efficiency and adaptability for practical online cognitive electronic countermeasure systems.

## Figures and Tables

**Table 1:** Representative related works and their main characteristics.

Category	Representative Work	Main Characteristics	Few-Shot	Physics Prior	Causal Design
Traditional feature engineering	[4,9,10,11]	Handcrafted signal descriptors with conventional classifiers	×	✓	×
Deep learning for jamming recognition	[12,13,14,15]	Deep representation learning for complex jamming patterns	×	weak/implicit	×
Few-shot jamming recognition	[16,17,18,19,20]	Limited-sample adaptation and generalization	✓	limited	×
Causal intervention for limited-data radar recognition	[21]	Deconfounding-oriented intervention learning	partial	×	✓

**Table 2:** Echo signal and jamming parameter settings.

Type	Parameter	Value
	Pulse width	3μs
LFM	Bandwidth	20MHz
	Sampling frequency	25MHz
RMJ	Number of false targets	3–6
	Delay of false targets	1–3μs
VMJ	Number of false targets	3–6
	Doppler frequency shift	1–5MHz
C&I	Number of rectangular pulse trains	2–4
	Number of time slots filled per segment	2–4
ISRJ	Intermittent sampling pulse width	0.5–1μs
	Intermittent sampling period	0.75–1.5μs
	Intermittent sampling pulse width	0.5–1μs
ISCJ	Intermittent sampling period	0.75–1.5μs
	Number of repeat forwards	2–4
SMSP	Sampling multiplier	3–5
SNJ	Number of repeat forwards	2–4
	Noise bandwidth multiplier	0.5–1.5

**Table 3:** Per-class recognition accuracy (%) of different methods under 20-shot.

Class	WECNN-TL	MAML	JR-TFSAD	PSPNet	PG-VCIN
LFM	95.69 ± 3.91	97.24 ± 2.96	78.55 ± 8.87	95.51 ± 1.99	**99.76 ± 0.13**
RMJ	91.30 ± 6.84	98.59 ± 1.69	99.17 ± 0.91	99.34 ± 0.62	**100.00 ± 0.00**
VMJ	93.13 ± 4.41	93.36 ± 6.64	97.80 ± 2.41	99.31 ± 0.30	**100.00 ± 0.00**
C&I	87.43 ± 4.08	86.88 ± 9.59	74.79 ± 17.41	93.91 ± 3.12	**96.69 ± 0.96**
ISRJ	85.02 ± 7.36	85.84 ± 13.68	**98.54 ± 0.68**	82.70 ± 9.67	95.84 ± 2.76
ISCJ	82.18 ± 10.72	60.57 ± 12.25	51.35 ± 20.73	83.86 ± 2.70	**97.42 ± 0.89**
SMSP	87.10 ± 4.49	93.79 ± 5.78	**99.56 ± 0.48**	94.36 ± 2.32	98.81 ± 0.46
SNJ	72.59 ± 15.33	56.53 ± 18.53	44.58 ± 15.84	83.38 ± 4.33	**92.09 ± 2.38**
RMJ*	**100.00 ± 0.00**	99.30 ± 0.37	**100.00 ± 0.00**	99.60 ± 0.20	**100.00 ± 0.00**

The best results are shown in bold, and the second-best results are underlined.

**Table 4:** Comparison of average recognition performance under 20-shot.

Metric (%)	WECNN-TL	MAML	JR-TFSAD	PSPNet	PG-VCIN
Accuracy	88.59 ± 3.04	85.80 ± 1.74	82.67 ± 2.13	92.07 ± 1.65	**97.77 ± 0.29**
Recall	89.15 ± 2.92	86.22 ± 1.88	84.29 ± 1.14	92.47 ± 1.09	**97.76 ± 0.28**
Precision	88.38 ± 3.29	85.06 ± 2.09	81.65 ± 1.91	92.27 ± 1.37	**97.75 ± 0.29**
F1-score	88.27 ± 3.30	85.87 ± 2.21	82.71 ± 1.88	92.07 ± 1.65	**97.82 ± 0.25**

The best results are shown in bold, and the second-best results are underlined.

**Table 5:** Comparison of average recognition performance under 10-shot.

Metric (%)	WECNN-TL	MAML	JR-TFSAD	PSPNet	PG-VCIN
Accuracy	83.67 ± 2.82	81.86 ± 2.48	80.17 ± 2.14	88.44 ± 1.42	**94.91 ± 0.51**
Recall	84.62 ± 3.55	83.24 ± 4.01	81.67 ± 2.53	86.48 ± 1.41	**94.86 ± 0.50**
Precision	84.05 ± 3.05	81.87 ± 2.40	80.91 ± 2.00	85.72 ± 1.48	**94.81 ± 0.51**
F1-score	83.92 ± 3.33	82.08 ± 1.19	80.46 ± 2.01	86.10 ± 1.68	**94.85 ± 0.51**

The best results are shown in bold, and the second-best results are underlined.

**Table 6:** Comparison of average recognition performance under 5-shot.

Metric (%)	WECNN-TL	MAML	JR-TFSAD	PSPNet	PG-VCIN
Accuracy	81.73 ± 3.02	72.87 ± 5.17	76.03 ± 4.39	83.50 ± 1.47	**91.84 ± 0.60**
Recall	84.70 ± 2.85	76.16 ± 4.93	72.89 ± 4.36	83.61 ± 2.52	**91.90 ± 0.46**
Precision	81.18 ± 3.03	72.91 ± 5.00	76.01 ± 3.83	83.50 ± 1.70	**91.92 ± 0.59**
F1-score	79.56 ± 3.09	72.55 ± 5.29	70.90 ± 4.80	83.55 ± 1.42	**92.31 ± 0.50**

The best results are shown in bold, and the second-best results are underlined.

**Table 7:** Complexity comparison of different methods.

Metric	WECNN-TL	MAML	JR-TFSAD	PSPNet	PG-VCIN
FLOPs ($\times 10^{9}$)	36.03	0.30	11.98	0.09	0.32
Params ($\times 10^{6}$)	20.82	11.18	18.37	0.20	0.32

**Table 8:** Ablation results of key modules under 20-shot.

Physics-Guided	VIB	Accuracy (%)	Recall (%)	Precision (%)	F1-Score (%)
×	×	91.84 ± 0.66	91.20 ± 0.60	91.66 ± 0.67	91.31 ± 0.68
×	✓	95.39 ± 0.40	95.41 ± 0.35	95.44 ± 0.39	95.37 ± 0.41
✓	×	94.81 ± 0.44	94.85 ± 0.45	94.91 ± 0.45	94.84 ± 0.44
✓	✓	97.77 ± 0.29	97.76 ± 0.28	97.75 ± 0.29	97.84 ± 0.25

**Table 9:** Sensitivity analysis of the VIB regularization coefficient β under the 20-shot setting.

β	Accuracy (%)	Recall (%)	Precision (%)	F1-Score (%)
$10^{-4}$	95.87 ± 0.46	96.06 ± 0.43	95.92 ± 0.46	95.89 ± 0.47
$5\times 10^{-4}$	97.28 ± 0.37	97.30 ± 0.30	97.28 ± 0.36	97.28 ± 0.37
$10^{-3}$	97.77 ± 0.29	97.76 ± 0.28	97.75 ± 0.29	97.82 ± 0.25
$5\times 10^{-3}$	96.94 ± 0.46	97.14 ± 0.43	97.00 ± 0.46	96.96 ± 0.46
$10^{-2}$	96.39 ± 0.60	96.54 ± 0.46	96.44 ± 0.59	96.41 ± 0.50

**Table 10:** Average recognition performance of PG-VCIN under compound-jamming conditions in the supplementary 20-shot experiment.

Method	Accuracy (%)	Recall (%)	Precision (%)	F1-Score (%)
PG-VCIN	97.20 ± 0.20	97.23 ± 0.19	97.21 ± 0.20	97.20 ± 0.21

## Data Availability

The data presented in this study are available upon request from the corresponding author. The data are not publicly available due to the fact that they currently include privileged information.

## Abbreviations

The following abbreviations are used in this manuscript:

PG-VCIN	Physics-Guided Variational Causal Intervention Network
VIB	Variational Information Bottleneck
FiLM	Feature-wise Linear Modulation
STFT	Short-Time Fourier Transform
DRFM	Digital Radio Frequency Memory
LFM	Linear Frequency Modulation
RMJ	Range Multi-False-Target Jamming
VMJ	Velocity Multi-False-Target Jamming
C&I	Chopping and Interleaving
ISRJ	Interrupted Sampling Repeater Jamming
ISCJ	Interrupted Sampling and Cyclic Repeater Jamming
SMSP	Smeared Spectrum Jamming
SNJ	Smart Noise Jamming
SNR	Signal-to-Noise Ratio
JSR	Jammer-to-Signal Ratio
KL	Kullback–Leibler
AWGN	Additive White Gaussian Noise
FrFT	Fractional Fourier Transform
FLOPs	Floating-Point Operations
IRM	Invariant Risk Minimization
SAR	Synthetic Aperture Radar
ATR	Automatic Target Recognition
